# Attenuated neuroprotective effect of riboflavin under UV-B irradiation via miR-203/c-Jun signaling pathway *in vivo* and *in vitro*

**DOI:** 10.1186/1423-0127-21-39

**Published:** 2014-05-07

**Authors:** Amit Kumar Tripathi, Ashish Dwivedi, Manish Kumar Pal, Namrata Rastogi, Priyanka Gupta, Shakir Ali, Manjunatha Prabhu BH, Hari Narayan Kushwaha, Ratan Singh Ray, Shio Kumar Singh, Shivali Duggal, Bhaskar Narayan, Durga Prasad Mishra

**Affiliations:** 1 Division of Endocrinology, CSIR-Central Drug Research Institute, Jankipuram Extension, Lucknow 226031, India; 2 Photobiology Division, Indian Institute of Toxicology Research, MG Marg, Lucknow 226001, India; 3 Department of Obstetrics and Gynaecology, KGMU, Lucknow 226003, UP, India; 4 Department of Biochemistry, Jamia Hamdard (Hamdard University, New Delhi, India; 5 Pharmacokinetics and Metabolism Division, CSIR-Central Drug Research Institute, Lucknow 226031, India; 6 Department of Meat, Fish & Poultry Technology, CSIR-Central Food Technological Research Institute, Mysore 570 020, India

**Keywords:** Cerebral ischemia, Riboflavin, UV-B, miR-203, C-jun, Neuroprotection

## Abstract

**Background:**

Riboflavin (RF) or vitamin B2 is known to have neuroprotective effects. In the present study, we report the attenuation of the neuroprotective effects of RF under UV-B irradiation. Preconditioning of UV-B irradiated riboflavin (UV-B-RF) showed attenuated neuroprotective effects compared to that of RF in SH-SY5Y neuroblostoma cell line and primary cortical neurons *in vitro* and a rat model of cerebral ischemia *in vivo*.

**Results:**

Results indicated that RF pretreatment significantly inhibited cell death and reduced LDH secretion compared to that of the UV-B-RF pretreatment in primary cortical neuron cultures subjected to oxygen glucose deprivation *in vitro* and cortical brain tissue subjected to ischemic injury *in vivo*. Further mechanistic studies using cortical neuron cultures revealed that RF treatment induced increased miR-203 expression which in turn inhibited c-Jun expression and increased neuronal cell survival. Functional assays clearly demonstrated that the UV-B-RF preconditioning failed to sustain the increased expression of miR-203 and the decreased levels of c-Jun, mediating the neuroprotective effects of RF. UV-B irradiation attenuated the neuroprotective effects of RF through modulation of the miR-203/c-Jun signaling pathway.

**Conclusion:**

Thus, the ability of UV-B to serve as a modulator of this neuroprotective signaling pathway warrants further studies into its role as a regulator of other cytoprotective/neuroprotective signaling pathways.

## Background

Cerebral stroke, a leading cause of death and disability worldwide involves cerebral ischemia-reperfusion injury and impaired blood flow resulting in neuronal cell death [1,2]. Despite the recent efforts for improvement of treatment strategies for cerebral stroke, prognosis of cerebral ischemia patients has remained largely unsatisfactory. This is attributed to the lack of effective neuroprotective agents for salvaging neuronal cell death as in most cases only the recombinant tissue plasminogen activator (rtPA) is routinely used for treatment [2]. Therefore, the need for expedited development of effective neuroprotective agents for cerebral stroke is critical.

Understanding of the complex pathophysiology of ischemic stroke is imperative for identifying promising neuroprotective agents and therapeutic strategies [2]. Recent studies have established microRNAs (miRs) as novel regulators of brain function with roles in cerebral ischemia and injury, neuroprotection, and neurodegeneration [3-7].

At current rates, it takes a reasonably long period of time for a lead compound to be developed into a clinically approved drug for cerebral ischemia [2,3]. An arguably faster path to development is to repository dietary agents or neutraceuticals as neuroprotective agents for improvement of cerebral stroke outcome [8]. The insufficiency of vitamins and antioxidants leads to increased cognitive injury in stroke patients [9,10]. In particular, riboflavin (vitamin B2) is known to have promising neuroprotective effects [9-12]. However, RF contains a photosensitive isoalloxazine ring making it vulnerable to the atmospherically predominant UV-B radiation (280-315 nm) induced photodegradation, which might compromise its neuroprotective effects [13-15]. In the present study, we examined the neuroprotective effects of RF and UV-B-RF in a rat model of cerebral ischemia and cortical neurons and investigated the molecular mechanisms involved in this process.

## Methods

The human neuroblastoma SH-SY5Y was obtained from American Type Culture Collection (ATCC, Rockville, MD). Riboflavin, para-formaldehyde, sucrose, poly-l-lysine, 2, 3, 5-triphenyl tetrazolium chloride (TTC), Nembutal, 4′, 6-diamidino-2-phenylindole (DAPI) all are procured from Sigma (St. Louis, MO, USA). The firefly luciferase reporter plasmid (Genecopeia Inc., Rockville, MD, USA) and the miRNA mimic was procured from Ambion (Ambion, Austin, TX, USA). The Lipofectamine 2000 and RNAiMax were from Invitrogen (Invitrogen, Carlsbad, CA,USA). The dual luciferase assay kit was purchased from Promega (Promega, Madison, WI, USA). A 3–0 rounded-tip nylon monofilament suture was procured from Ethicon. The cell counting kit-8 (CCK-8) was from Dojinando Laboratory (Dojindo Molecular Technologies, Inc. Rockville, MD,USA). The RT^2^miRNA PCR array system was procured from Qiagen (Qiagen, Hilden, Germany). The primary antibodies (p-H2AX (Cat No 9718), Cleaved Casapse-3 (Cat No 9661), c-Jun (Cat No 9165), and Beta Actin (Cat No 4967) were procured from the Cell Signaling Technology (Cell Signaling Technologies, Boston, MA, USA) The secondary antibodies used in the experiments were from Chemicon (Chemicon, Temecula, CA, USA). All other chemicals were purchased from Sigma (St. Louis, MO, USA) unless otherwise stated.

### UV-B irradiation

The UV-irradiation system (Vilber Lourmat, France), was equipped with calibrated UV-B detection probe and comprised of an array of 1.2 m long UV-B emitting tubes for regulated emission of radiation through a microprocessor-controlled RMX-3 W radiometer. The spectral emission of UV-B source used for the experiments ranged from 280 to 320 nm with a peak at 315 nm. Intensity of UV-B (0.6 mW/cm^2^ for 1 h) selected for irradiation was based on dosimetry carried out between 12.00 Noon to 1.00 PM and was parallel to the ambient intensity of UV-B radiation in sunlight at Lucknow (26°45′N latitude and 80°50′E longitude at 146 m above the mean sea level). UV-B irradiation was carried out in a temperature controlled (25°C ± 2°C) radiation chamber. The riboflavin samples in glass Petri dishes (60 × 15 mm) were placed at a minimum distance of 22.0 cm from the source of radiation.

### Liquid chromatography-mass spectrometry (LC-MS/MS) analysis

Mass spectrometric detection was performed on an API 4000 QTRAP mass spectrometer (Applied Biosystems, Canada). RF was optimized by continuous infusion at 10 μl min^−1^ using syringe pump (Model ‘11’, Harvard apparatus). Zero air and nitrogen gas were used as source and curtain gas, respectively. The optimized declustering potential was 120 Volt. At these optimized conditions, Q1 scan for control and test samples was performed.

### Cell culture and transfections

SH-SY5Y cells were maintained plated in 100-mm cultured dishes and cultured in Eagle’s modified essential medium/ F12, supplemented with 10% fetal calf serum, 1% of a mixture of penicillin/streptomycin/nystatin, 1 mM sodium piruvate, 0.1 Mm non-essential amino acids,1.5 g/L sodium bicarbonate and 2 mM L-Glutamine. RF was dissolved in saline (0.9% NaCl) and stock solutions (10 mM) were further diluted in the culture media prior to the use in experiments. The cells were plated at 1×10^4^cells per well in 96-well or 5×10^5^cells per well in 6 well microtiter plates for the assays. For the primary cortical neuronal cultures, embryonic day 16–18 pups were obtained from pregnant Sprague Dawley rats, anesthetized with tribromoethanol (350 mg/kg, i.p.). Meninges were carefully removed and isolated cerebral cortices were dissociated with 8.2 U/ml papain (Worthington Biochemical, Lakewood, NJ) for 30 min at 37°C in a shaking water bath. Subsequently, fetal bovine serum and trypsin inhibitor were used to stop digestion. The tissue suspension was then triturated thoroughly using a pasteur pipette. Freshly dissociated cells were seeded at 2 × 10^5^ cells/cm^2^ into 96-well plastic plates coated with L-polyornithine (10 μg/ml) and then incubated in Neurobasal medium (Invitrogen, Carlsbad, CA) with 2% B-27 supplement, Glutamax (1:100) (Invitrogen, Calsbad, CA), penicillin, and streptomycin at 37°C with 5% CO_2_ and 95% air. The medium was changed 24 h after plating, and half of the medium was changed every 3 days. Experiments were conducted after three changes of media. Immunocytochemical analysis of neuronal marker protein gene product 9.5 (PGP 9.5) (Chemicon International, Inc., Temecula, CA) was used to confirm the purity of neuronal cells. The transfections were carried out by using either Lipofectamine 2000 or RNAiMax (Invitrogen, Carlsbad,CA,USA) as per a standardized protocol [16].

### Measurement of cell viability and LDH secretion

The cell viability and LDH secretion were quantified in the SH-SY5Y cell line and cortical neuron cultures using the Cell Counting Kit-8 (Dojindo, Molecular Technologies, MD, USA) and the LDH quantification kit (Biovision, CA, USA) as per the manufacturer’s instructions.

### Oxygen glucose deprivation

For oxygen glucose deprivation (OGD) experiments, the media of cultured SH-SY5Y cell line or cortical neurons were replaced with pre gassed 1X Hank’s balanced salt solution (HBSS, 140 mM NaCl, 5 mM KCl, 2 mM CaCl_2_, 10 mM HEPES, 30 μM glycine, pH 7.4) and placed in a Billups-Rothenberg modular incubator chamber (Del Mar, CA) and flushed with a gas mixture of 5% CO_2_ and 95% N_2_ for 10 min. The chamber was then sealed and placed into a humidified CO_2_ incubator at 37°C. After 60 min in the hypoxic chamber, the OGD treatment was stopped by replacing HBSS with the respective cell culture media. The cells were then placed back to normoxic conditions and incubated for 24 h for the functional assays.

### Focal cerebral ischemia and neurological deficit score evaluation

Focal cerebral ischemia was simulated in a rat model of cerebral stroke through the middle cerebral artery occlusion (MCAO). Adult male Sprague–Dawley (SD) rats (220 ± 20 g) were obtained from the National Laboratory Animal Centre, Central Drug Research Institute (CDRI), Lucknow, used for experiment. The experimental animals were approved by Institutional Animal Ethical Committee (IAEC) and all animal experiments were carried out in accordance with the institutional guidelines. Rats were housed in cages in a temperature-controlled (25°C ± 1°C) environment, provided free access to food and purified drinking water ad libitum. The rats were divided into 4 groups of 6 rats each as follows: Group I: Sham operated group; handled as other groups, except MCAO was not done. Group II: Ischemic brain damage induced by MCAO, treated with saline as vehicle. Group III: Ischemic brain damage, treated with 10 mg/kg of RF 30 min before MCAO. Group IV: Ischemic brain damage, treated with 10 mg/kg UV-B irradiated RF 30 min before MCAO. The induction of MCAO and evaluation of the neurological deficit score was conducted as per a standardized protocols [17,18]. At the end of the experimental period the animals were sacrificed through decapitation.

### Western blotting

Western blotting experiments were carried out using previously standardized protocol [16]. Briefly both cells and tissues lysates were prepared in cell lysis buffer (50 mMol/L Tris–HCl, 150 mmol/L NaCl, 1% NP40, 0.5% SDS, and 1% deoxycholic acid). The lysates were subsequently heated at 95°C for 5 minutes and centrifuged at 14,000X g for 5 min and the supernatants were stored at −80°C until use. 50 μg of estimated protein per sample was loaded on to 10% SDS-PAGE gels. Blocking was done with 2% BSA and blots were incubated in primary antibodies (1:5000) over night at 4°C. The blots were washed thrice in 0.1% Tween-20 in PBS and incubated with HRP-conjugated secondary antibody (1:2000) for 1 h at room temperature. Blots were developed using the chemiluminescent substrate (Millipore, Billerica, CA, USA).

### MiRNA analysis

Total miRNA was isolated from the cortical tissues samples and cortical neuronal cultures using the Nucleospin miRNA kit (Macherey–Nagel, Duren, Germany). The change in miRNA expression was measured using the RT^2^ miRNA PCR array system (Qiagen, Hilden, Germany**)**. Expression analysis of 376 miRNA sequences was performed as per the manufacturer’s instructions in a Light Cycler 480 II (Roche Diagnostics, Mannheim, Germany). The PCR conditions were set according to the manufacturer’s instructions. Data analysis was performed using the RT^2^ Profiler PCR Array Data Analysis Template **(**Qiagen, Hilden, Germany**)**. Normalization of the data was done using four miRNAs (hsa-SNORD-44, hsa-SNORD47, hsa-SNORD48 and hsa-U6) and the relative miRNA expression levels were calculated with 2^-ΔΔCt^. All the experiments were performed in triplicate.

### Site directed mutagenesis and real-time PCR

The expression vector of c-Jun deletion mutant N1–220 was generated in pcDNA3.1 vector by PCR. The expression vectors of c-Jun mutant (pcDNA3.1junS63/73A and pcDNA3.1junM3A) were generated in pcDNA3.1 vector by PCR using a previously standardized site-directed mutagenesis method [19]. All generated constructs were verified by sequencing.

### Real-time PCR analysis

mRNA from the samples was extracted using Trizol. To evaluate the level of c-Jun expression, realtime PCR with SYBR Green dye was used in a LC480 II light cycler real time PCR machine. The real time PCR reaction mixture contained 10 μl Syber Green Super Mix, 100 nM of each primer (c-Jun Forward Primer: 3′- TGATGACGCCTTACGTGGTA-5′ and Reverse Primer:3′- ACAAGGTGTTCCGAGCTGTT-5′) and 1 μl cDNA. All samples were run in triplicates and each experiment was repeated at least three times independently. Each sample was normalized on the basis of GAPDH.

### Luciferase assay

The luciferase reporter plasmids containing the wild-type 3′UTR with the miR-203 binding site of c-Jun was obtained from Genecopoeia (Rockville, MD, USA). respectively. Cortical neuron cells were transfected with the luciferase constructs (100 ng per 24-well) and pre-miR-203 or pre-miR-Control (10 nM, Applied Biosystems) using Lipofectamin 2000 (Invitrogen). Luciferase activity was measured after 24 h using the Dual Luciferase Reporter Assay according to the manufacturer’s instructions (Promega, Madison, WI, USA).

### Statistical analysis

All the values are represented as mean ± SEM from at least three independent experiments. Data was analysed using One-way ANOVA followed by Newman Keuls multiple comparision test. Values with p < and =0.05 were considered to be significant.

## Results

### UV-B irradiation induces degradation of RF and decreases its neuroprotective effects *in vitro*

**Figure 1:**
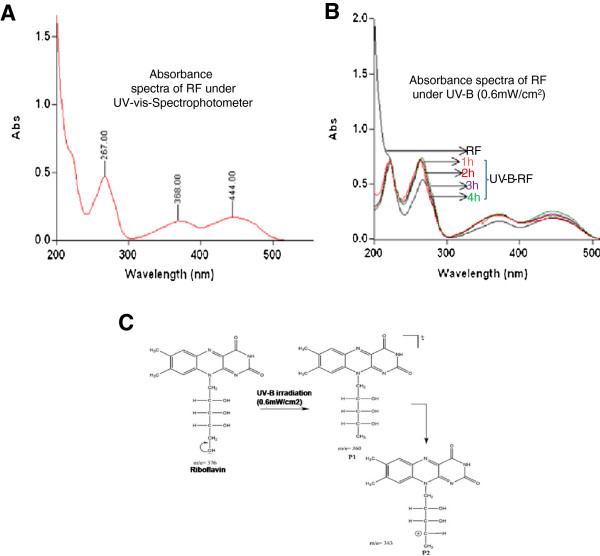
**Absorbance spectra of RF under UV-B by UV–vis-Spectrophotometer. (A)** Absorbance spectra of RF (5 μg/ml) showed maximum absorbance in UV-B region. **(B)** Absorbance spectra of UV-B-RF in different time interval (1 to 4 h) compared to RF. **(C)** Schematic representation of RF photodegradation and photoproducts ion formation under UV-B (0.6 mW/cm2 for 1 h) irradiation, identified by LC-MS/MS analysis. Representative data of three independent experiments.

**Figure 2:**
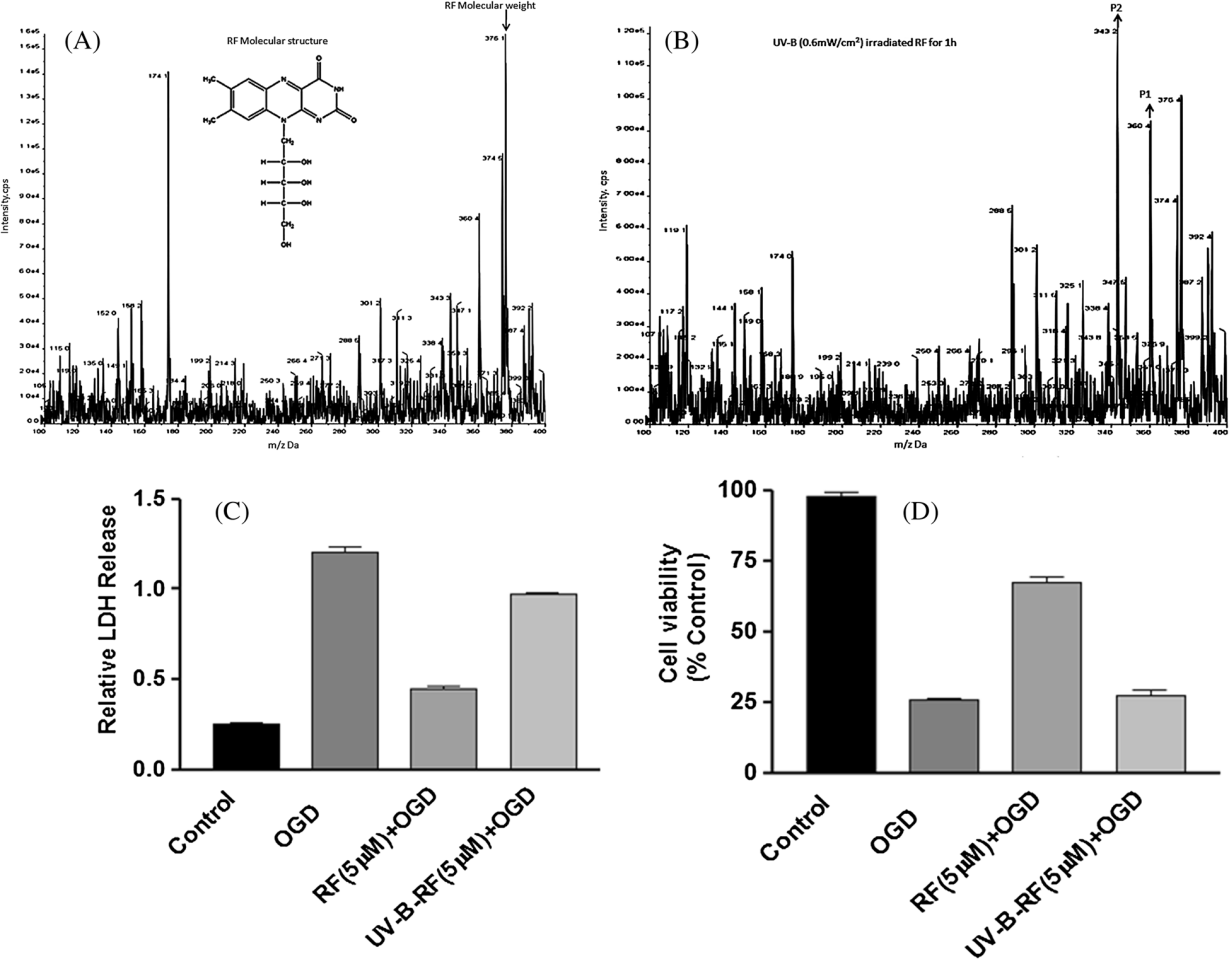
**UV-B irradiation induces photodegradation and attenuation of the neuroprotective effects of RF in human neuroblostoma SH-SY5Y cell lines. (A)** LC-MS/MS spectrum of parent RF (376 Da) and **(B)** two major photoproducts P1 (360.4 Da) and P2 (343.2 Da) after UV-B (0.6 mW/cm 2 for 1 h) irradiation. **(C)** RF treatment significantly inhibits OGD induced LDH release compared to that of UV-B-RF treatment **(D)** RF treatment significantly rescues cell viability compared to that of UV-B-RF treatment in Human neuroblostoma SHSY5Y cell line. The average of three independent experiments performed in triplicate is shown. Statistical significance was analyzed by ANOVA. Values are the mean ± SEM *p < 0.05 and **p < 0.01 vs. RF treated and control group.

RF is known to exert neuroprotective effects [9-12] and is vulnerable to photodegradation [13-15]. We therefore assessed the effects of UV-B irradiation induced photodegradation of RF. The absorption spectrum of RF (5 μg/ml) in physiological saline at different intensities (Figure 1A) is characterized by one intense absorption peak at 267 nm in the UV-B region. The photodegradation study was performed under UV-B (0.6 mW/cm^2^) for 1, 2, 3 and 4 h. The result showed 1 h irradiation of RF shifted the absorption maxima towards the UV range (Figure 1B, C). These results were further confirmed through the LC-MS/MS analysis. LC-MS/MS (Q1 scan) analysis was performed with RF (376 Da) and UV-B irradiated RF (Figure 2 A, B). Two major photoproducts were obtained with molecular mass of P1 (360.4 Da) and P2 (343.2 Da), respectively. These two ionic photoproducts formed by elimination of hydroxyl group after UV-B irradiation. In LC-MS/MS spectra of RF, photoproduct ion formed at collision energy was found to be different from photoproduct of UV-B irradiated sample (Figure 1C). Next, we explored whether the photodegradation of RF influenced its neuroprotective effects. We subjected SH-SY5Y cells to oxygen glucose deprivation/reperfusion (OGD/R) and assessed the neuroprotective effects of UV-B-RF and RF through quantification of the LDH release and cell viability. The results indicated that OGD significantly increased cellular stress indicated by the increased (p < 0.01) LDH secretion (Figure 2C) in control cells. Pre-treatment of the SH-SY5Y cells with RF (5 μM) before OGD reduced the OGD-induced LDH release by 46.2% while in the UV-B-RF treated group the reduction of LDH secretion was only 15.9% (Figure 2C). Similarly, OGD significantly (p < 0.01) reduced (~23.8%) the cell viability in the control group. Treatment of SH-SY5Y cells with RF significantly (p < 0.01) increased cell viability (~70.3%) compared to that of the UV-B-RF treatment (~24.2%) (Figure 2D). Collectively, these results showed that the UV-B irradiation decreased the cytoprotective effect of RF in the human neuroblostoma SH-SY5Y cell line *in vitro*.

### UV-B-RF has attenuated neuroprotective effects in a rat model of cerebral ischemia

**Figure 3:**
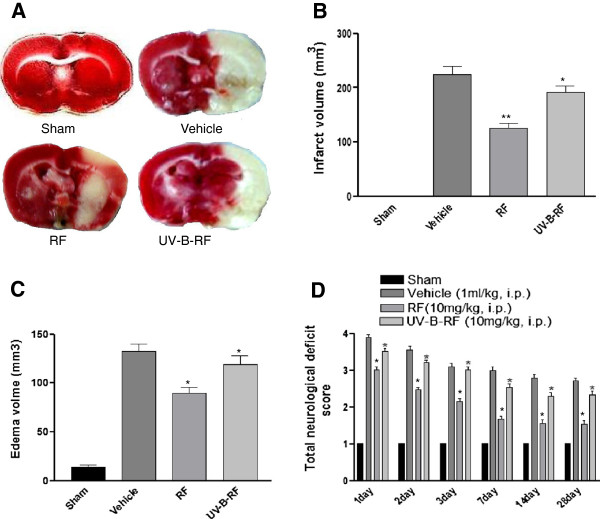
**UV-B irradiation reduces the neuroprotective effects of RF in a rat model cerebral ischemia. (A)** TTC staining after 24 h reperfusion of single coronal brain section treated with either RF or UV-B-RF before MCAO. **(B, C)** Effect of pretreatment with vehicle, RF and UV-B-RF on infarct volume (mm^3^) and Edema volume (mm^3^) calculated from image-J analysis software. **(D)** Total neurological deficit score was evaluated to see neurological impairment after 1 to 28 days successive treatment. Statistical significance was analyzed by ANOVA. Values are the mean ± SEM.*p < 0.05 and **p < 0.01 vs. RF treated and control group.

**Figure 4:**
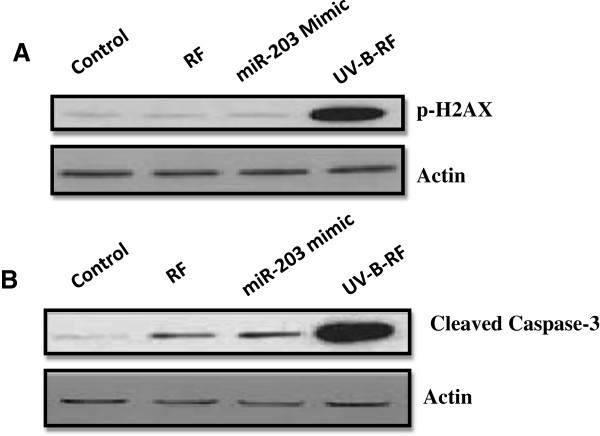
**RF and UV-B-RF have differential effects on DNA damage and caspase-3 activation in the cortical region of brain subjected to ischemic injury.** Immunoblotting analysis of **(A)** p-H2AX and **(B)** Cleaved caspase-3 in cell lysates prepared from the cortical regions of the brain subjected to ischemic injury and pretreated with either vehicle (saline), RF (10 mg/kg i.p.), UV-B-RF(10 mg/kg, i.p.) or miR-203 mimic (200 nM/kg i.p.). Representative images of at least three independent experiments are shown.

RF is known to have neuroprotective effects in a rat model of cerebral ischemia [10,12]. As our results indicated attenuated neuroprotective effects of UV-B-RF *in vitro*, we next tried to assess, the neuroprotective effects of UV-B irradiation in a rat model (middle cerebral artery occlusion) of cerebral ischemia. The results showed that RF treatment (10 mg/kg body weight, i.p.) showed significant neuroprotective activity compared to that of the UV-B-RF (10 mg/kg body weight, i.p.) as indicated by the decreased neuronal cell death in single coronal brain section (Figure 3A) and p-H2AX and Caspase-3 immunobloting analysis of brain lysates (Figure 4A,B). UV-B-RF treatment also failed to reduce the infract volume (Figure 3B) and edema volume (Figure 3C). Further, the evaluation of the neurological deficit scores (on days 1, 2, 3, 7, 14 and 28) clearly indicated that RF treated experimental groups had significantly improved neurological scores compared to that of the of the UV-B-RF treated groups (Figure 3D). These results collectively suggested that UV-B-RF had attenuated neuroprotective effects in a rat model of cerebral ischemia *in vivo*.

### UV-B irradiation differentially regulates RF induced expression of miR-203 in-vivo and in-vitro

**Figure 5:**
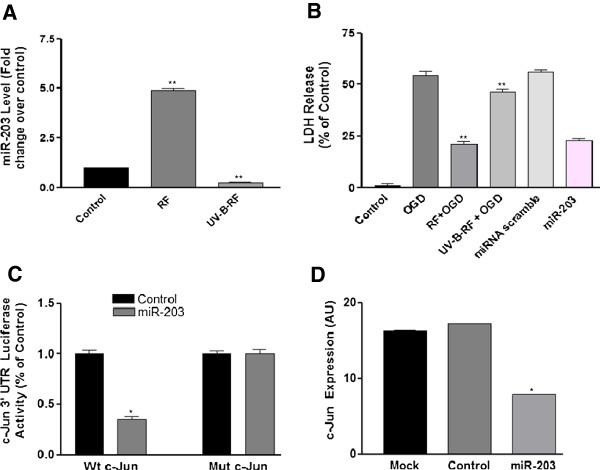
**UV-B irradiation differentially regulates RF induced expression of miR-203 in vivo and in vitro. (A)** Cortical neuronal cells were subjected to OGD were treated with either RF (5 μM) or UV-B-RF (5 μM). RF significantly increased the expression of miR-203 compared to that of UV-B-RF **(B)** RF treatment or miR-203 overexpression significantly decreased the LDH secretion compared to that of UV-B-RF treatment. **(C)** Percentage c-Jun 3′ UTR luciferase activity assay of wild type and mutated c-Jun in cortical neuron pre-treated with vehicle control and miR-203. **(D)** c-Jun expression in cortical neuron subjected to OGD with pre-treatment of mock, Control and miR-203. The average of three independent experiments performed in triplicate. Statistical significance was analyzed by ANOVA. Values are the mean ± SEM. *p < .05 and **p < 0.01 vs. RF treated and control group.

Experimental evidence has established the role of miRNAs as critical regulators of neuronal cell survival and cell death [3-7]. As the earlier results indicated that the UV-B irradiation decreased the neuroprotective ability of RF *in vivo*, we next tried to assess the possible role of miRNAs involved in this process. The miRNA PCR array analysis indicated that the RF preconditioning increased expression of miR-203, whereas UV-B-RF treatment induced only 0.25 and 0.50 fold change in its expression in cortical neuron cells and cortical brain tissue respectively (Table 1). We further tried to confirm this finding using primary cortical neuron cultures. The results indicated that RF treatment significantly increased the miR-203 levels (Figure 5A) compared to that of UV-B-RF treatment (Table 1). Similarly, RF treatment significantly decreased the OGD induced LDH secretion, compared to that of the UV-B-RF treatment (Figure 5B). This finding was further confirmed by the comparable reduction in LDH secretion through miR-203 over expression in cortical neurons (Figure 5B). These results clearly indicated that, RF treatment sustained the increased expression of miR-203 in neuronal cells while UV-B-RF has no significant effect on its expression. Collectively, these results showed that UV-B irradiation alters the RF induced increase in the expression of miR-203 in neuronal cells both *in vivo* as well as *in vitro*.

**Table 1:** Effect of RF and UV-B-RF treatment on differential expression of miRNAs cerebral cortex in vivo and cortical neuronal culture in vitro

**Expression of miRNAs in rat cerebral cortex treated RF and UV-B-RF**				
**miRNAs**	**RF Pre- treatment (Fold Change)**	**P Value**	**UV-B irradiated RF-pre treatment (Fold Change)**	**P Value**
miR-203	3.50	0.05	0.50	0.05
miR-23a	1.10	0.30	1.20	0.25
miR-10b	1.20	0.40	1.10	0.30
miR-145	1.20	0.60	1.10	0.35
miR-350	1.30	0.45	1.20	0.50
miR-27a	0.90	0.64	0.80	0.49
**Expression of miRNAs in rat primary cortical neurons treated with RF and UV-B-RF**				
miR-203	4.9	0.05	0.25	0.05
miR-23a	-1.2	0.06	-0.80	0.25
miR-10b	-1.3	0.20	-1.10	0.30
miR-145	1.1	0.40	0.94	0.35
miR-350	1.4	0.35	1.10	0.50
miR-27a	-1.3	0.44	-1.20	0.49

miR-203 expression level increases significantly (p<.05) in cortical brain tissue and neuronal culture.

### UV-B irradiation alters the neuroprotective ability of RF through modulation of the miR-203/c-Jun signaling pathway

**Figure 6:**
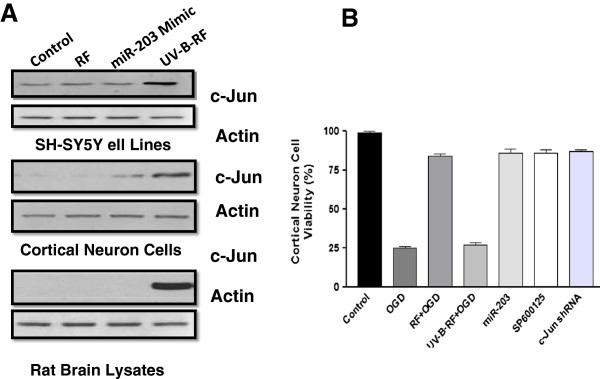
**UV-B irradiation modulates the miR-203/c-Jun signaling pathway for altering the neuroprotective effect of RF. (A)** Immunoblotting analysis of c-Jun expression in cell lysates from SH-SY5Y cell line and primary cortical neurons subjected to OGD and cortical regions of the brain subjected to ischemic injury and pretreated with either vehicle (saline), RF (10 mg/kg i.p.), UV-B-RF(10 mg/kg, i.p.) or miR-203 mimic (200 nM/kg i.p.). Representative images of at least three independent experiments are shown. **(B)** Cortical neuronal cells were subjected to OGD and treatment with RF (5 μM), miR-203 mimic (5 μM) , SP600125 (20 μM), c-Jun shRNA (50 nM/ml) and UV-B-RF (5 μM) treatment. Cell viability of cortical neurons was measured using the CCK-8 kit. UV-B-RF had significantly lower cell viability compared to that of the treated groups. The average of three independent experiments performed in triplicate. Statistical significance was analyzed by ANOVA. Values are the mean ± SEM. *p < 0.05 and **p < 0.01 vs. RF treated group.

The neuroprotective miR-203 is known to inhibit c-Jun [20-22] and the inhibition of c-Jun is known to have neuroprotective effects [23]. Therefore, we next explored the effects of increased miR-203 expression on c-Jun levels. Results of the luciferase assay indicated that the miR-203 targeted the 3′-UTR of c-Jun (Figure 5C). This was further confirmed through the over expression of miR-203, in cortical neurons which led to significant decrease in the c-Jun expression (Figure 5D). The evaluation of the direct effects of RF and UV-B-RF treatments on c-Jun expression revealed that RF or miR-203 overexpression significantly inhibited c-Jun protein expression in SH-SY5Y cells subjected to OGD and cortical neuron cells and cortical brain tissue subjected to ischemic injury, compared to that of the UV-B-RF treatment (Figure 6A). Finally we tried to confirm the role of the miR-203/c-Jun signaling on cortical neuron cell survival in the conditions of OGD. The results indicated that RF treatment significantly increased the neuronal cell survival during conditions of OGD compare to UV-B-RF. The RF induced increase neuronal cell survival was comparable to the over expression of miR-203, pharmacological and genomic inhibition of c-Jun in cortical neurons (Figure 6). Collectively, these results suggested that the UV-B irradiation alters the neuroprotective effects of RF through modulation of the miR-203/c-Jun signaling pathway.

## Discussion and conclusion

Riboflavin is a water-soluble, heat stable and light sensitive vitamin existing in a wide variety of foods [8,12]. Riboflavin has been reported to have neuroprotective effects through reduction of ischemic brain injury in focal cerebral ischemia [10,12]. The recent interest on the relationship of riboflavin and stroke is focused on the riboflavin deficiency in cerebral stroke patients [9] and the riboflavin regulation of circulating homocysteine concentrations, a risk factor for cardiovascular disease [8,12]. However, due to its light sensitive nature we hypothesized that the atmospherically predominant UV-B irradiation may alter the functional properties of RF and other phytochemicals such as piperine and curcumin [20] influencing its neuroprotective effects. Our study showed that UV-B irradiation significantly alters the neuroprotective effects of RF.

The miR-203 has neuroprotective effects [20] and it is known to regulate cell proliferation, differentiation and death [21,22]. Consistent with this finding, our study showed that the RF treatment in neuronal cells induced significantly increased expression of miR-203, decreased OGD induced LDH secretion and increased cell survival compared to that of the UV-B-RF treatment. We further identified c-Jun as a target of miR-203 and its inhibitory effect on c-Jun expression in agreement with earlier studies [21,22]. This may represent a critical step in the regulation of the neuroprotective effects of RF. The relative decrease in the miR-203 expression with respect to the controls in the cultured neuronal cells or brain tissues treated by UV-B-RF was 0.25 and 050 folds respectively. This finding may possibly due to the existence of a regulatory circuit, in which miR-203 and c-Jun mutually inhibit each other. This may represent a critical step in the neuroprotective action of RF. As c-Jun is a negative regulator of miR-203, the circuit constitutes a feedback loop, whereby RF treatment in neuronal cells had increased levels of miR-203 expression, inducing an inhibition of c-Jun resulting in neuroprotection [23,24], where as UV-B irradiated RF was devoid of this effect. Thus, our results suggest a novel notion: RF preconditioned neuronal cells have increased while UV-B-RF preconditioned neuronal cells have no marginal change in miR-203 expression thereby leading to differential effects on c-Jun inhibition and neuroprotection. Interestingly, we found RF treatment both in vivo and in vitro significantly increased miR-203 expression and subsequent c-Jun inhibition leading to neuroprotection. Further studies will be needed to understand whether this signaling pathway is a target of other neuroprotective or neurotoxic agents.

Taken together our studies suggest that (i) UV-B irradiation induces attenuation of the neuroprotective effects of RF (ii) through the modulation of the miR-203/c-Jun signaling pathway. Our current results strongly suggest that the potential of UV-B irradiation may be further investigated for its potential as a regulator of other cytoprotective/neuroprotective signaling pathways. Importantly, further studies are required to assess the translational value of the therapeutic activation of the miR-203/c-Jun signaling pathway for neuroprotection.

## Competing interests

The authors declare that they have no competing interests.

## Authors’ contributions

AKT, AD, MKP, NR, HK and PG carried out the experiments. DPM, SKS, SD, BN and RSR analyzed data. AKT, AD, and MKP designed the experimental protocols. AKT, DPM and AD prepared the manuscript. All authors read and approved the final manuscript.
